# Use of Positron Emission Tomography/Computed Tomography in Radiation Treatment Planning for Lung Cancer

**DOI:** 10.4274/mirt.19870

**Published:** 2016-06-06

**Authors:** Kezban Berberoğlu

**Affiliations:** 1 Anadolu Medical Center, Clinic of Nuclear Medicine, İstanbul, Turkey

**Keywords:** F18 fluorodeoxyglucose positron emission tomography/computed tomography, radiotherapy planning, non-small cell lung cancer, small cell lung cancer

## Abstract

Radiotherapy (RT) plays an important role in the treatment of lung cancer. Accurate diagnosis and staging are crucial in the delivery of RT with curative intent. Target miss can be prevented by accurate determination of tumor contours during RT planning. Currently, tumor contours are determined manually by computed tomography (CT) during RT planning. This method leads to differences in delineation of tumor volume between users. Given the change in RT tools and methods due to rapidly developing technology, it is now more significant to accurately delineate the tumor tissue. F18 fluorodeoxyglucose positron emission tomography/CT (F18 FDG PET/CT) has been established as an accurate method in correctly staging and detecting tumor dissemination in lung cancer. Since it provides both anatomic and biologic information, F18 FDG PET decreases inter-user variability in tumor delineation. For instance, tumor volumes may be decreased as atelectasis and malignant tissue can be more accurately differentiated, as well as better evaluation of benign and malignant lymph nodes given the difference in FDG uptake. Using F18 FDG PET/CT, the radiation dose can be escalated without serious adverse effects in lung cancer. In this study, we evaluated the contribution of F18 FDG PET/CT for RT planning in lung cancer.

## Abstract (tr)

Akciğer kanserlerinin tedavisinde radyoterapi önemli rol oynar. Küratif radyoterapinin uygun biçimde verilebilmesi için doğru tanı ve evrelendirme yapılması şarttır. Radyoterapi planlamada tümör sınırlarının doğru biçimde belirlenmesi ile coğrafik kayıpların önüne geçilebilir. Günümüzde radyoterapi planlamada bilgisayarlı tomografi (BT) görüntüleri kullanılarak tümör sınırları manuel olarak belirlenmektedir. Bu yöntem, kullanıcılar arasında tümör volümlerinin belirlenmesinde farklılıklara yol açmaktadır. Hızla gelişen teknoloji sayesinde radyoterapi cihazlarının ve yöntemlerinin değişmesi uygun tedavinin verilebilmesinde gerçek tümör dokusunun gösterilmesinin önemini arttırmıştır. F18 florodeoksiglukoz pozitron emisyon tomografi/BT’nin (F18 FDG PET/BT) akciğer kanserlerinde doğru evreleme ve tümör yayılımı tespit etmede güvenilir bir yöntem olduğu bildirilmiştir. F18 FDG PET anatomik bilgilere ilave olarak biyolojik bilgileri de sağladığından klinisyenler arasında farklılığı anlamlı biçimde azaltmaktadır. Örneğin tümör dokusunu atelektazik alandan ayırarak ve boyutları benign nedenlerle büyümüş lenf nodlarını belirleyerek tümör volümlerini azaltmakta veya boyutları küçük ancak FDG tutulumu gösteren metastatik lenf nodlarını göstererek doğru alanın ışınlamasına olanak sağlamaktadır. F18 FDG PET/BT ile akciğer kanserlerinde ciddi yan etkilere yol açmadan doz artırımı yapılabilir. Bu yazıda akciğer kanserlerinde radyoterapi planlamasının F18 FDG PET/BT ile yapılmasının katkısı değerlendirilmiştir.

## INTRODUCTION

External beam radiotherapy (RT) plays an important role in the management of non-small cell lung cancer (NSCLC) and small cell lung cancer (SCLC) (1,2). In the delivery of RT with curative intent, an optimum treatment plan will deliver a sufficiently high dose of radiation to achieve high tumor control while delivering the least possible dose to the smallest possible volume of critical normal tissues to reduce the side effects of RT. The introduced RT techniques such as three-dimensional conformal RT (3D-CRT), intensity modulated radiation therapy (IMRT) (3), and image-guided radiation therapy (IGRT) have improved the accuracy of radiation delivery, leading to improved loco-regional control with reduced morbidity by facilitating delivery of a higher radiation dose to the tumor while sparing more normal tissue (4,5). Before a treatment decision is made, accurate diagnosis and staging are essential parts of the RT treatment plan. The staging and diagnosis of the disease also play a crucial role in the success of definitive RT. Thus, feasible systemic treatments are enabled instead of unnecessary local treatments in patients with distant metastasis owing to accurate staging. The effectiveness of radiation therapy for lung cancer definitive treatment is limited by the radio-sensitivity of surrounding normal structures, by the difficulty in delineating the extent of malignant tissue using conventional imaging techniques, and by the identification of distant metastatic disease. Accurate and precise target delineation is necessary in order to take full advantage of these modern RT techniques. In addition, surgical resection is the standard of care for stage I and II NSCLC; however, significant co-morbidities may preclude surgical resection in those who are not able to tolerate the procedure. There is emerging data on the potential of ablative RT, called the stereotactic body radiation therapy (SBRT), in an effort to reduce morbidity and achieve better local control in a select group of patients. Herein we review the potential role of positron emission tomography (PET) imaging as a prognostic indicator in treatment planning and in the assessment of response to SBRT. Accurate delineation of target volume and preservation of peripheral critical organs determine treatment success in not only IMRT/volumetric modulated arc therapy but also SBRT. Target volumes and treatment volumes in RT planning are primarily determined by structural imaging with CT, contrast CT or MRI, which together with clinical judgment are used to estimate the likely extension of microscopic disease in each case and thereby define the clinical target volume (CTV). Currently computed tomography (CT) is still being used for the determination of tumor volume and to obtain electron density information that is necessary for organ dose calculation during treatment planning. In a clinical study where the tumor contour was drawn by radiation oncologists manually, there were inter-user differences between radiation oncologists in the determination of tumor volumes (6). Anatomic imaging methods can be insufficient for assessment of some tumors and lymph nodes, and it is observed that radiation field might not include the tumor despite radiation dose escalation (7). Feasibility studies have found that the use of F18 fluorodeoxyglucose-PET/CT (F18-FDG) for planning three-dimensional conformal radiation therapy improves the standardization of volume delineation as compared to CT alone in several types of cancers that are well imaged on PET (8). FDG-PET/CT was formed by the fusion of CT that allows anatomic information, and FDG-PET that provides biological information. Thus, both anatomic and biologic information is acquired together. FDG-PET/CT significantly decreases the delineation differences between oncologists, and it provides proper staging by identifying tumor and lymph nodes and hence determines gross target volume (GTV) more accurately during RT planning process. In addition, it was shown that PET/CT increases the sensitivity and accuracy in determining nodal GTVs than those detected by CT alone. The contribution of FDG-PET/CT to RT planning has been investigated in various cancer types (9). Currently, the widespread use of FDG-PET/CT allows RT planning by this method (10). Functional/biologic imaging by FDG-PET/CT changes RT planning due to several reasons. The most important ones are:

1. Detection of lesions that are not observed by CT and MR (identification of small lymph nodes and distant metastases),

2. Reduction of tumor volumes by determination of fields without tumor such as atelectasis,

3. Allowing dose heterogeneity within one target by determining biologic differences within the tumor,

4. Superior ability in assessing the tumor after chemo-RT and during treatment,

5. “Response modulated RT” planning based on changing target volumes during the course of treatment (11,12).

## RADIOTHERAPY PLANNING IN NON-SMALL CELL LUNG CANCER BY FLUORODEOXYGLUCOSE POSITRON EMISSION TOMOGRAPHY/COMPUTED TOMOGRAPHY

Lung cancer is the leading cause of cancer mortality. Five-year life expectancy is 14% in patients diagnosed with lung cancer, and surgery is indicated in approximately 1/3rd (13). Surgery is the primary treatment for patients with early-stage disease. However, RT plays a significant role in those who cannot be operated due to medical and technical reasons. Disease control can be achieved by delivering the maximum radiation dose to the tumor and decreasing the dose to peripheral tissues; in NSCLC, this can be achieved via novel RT techniques such as IMRT, IGRT, and SBRT. RT planning by CT is a standard approach. Planning is performed by using anatomic information obtained by CT. When PET is used, biologic data can be included in planning as well, which allows dose escalation to the GTVs.

## STAGING

Staging is the most critical process in NSCLC, since both treatment strategy and prognosis are subject to change according to disease stage. FDG-PET/CT is more accurate than CT in showing mediastinal and distant metastases, and this imaging platform changes the treatment plan in approximately 1/3rd of patients (14). The sensitivity of FDG-PET/CT in mediastinal staging is higher than CT due to increased metabolic uptake of lymph nodes in FDG-PET/CT that were otherwise noted to be of normal size by CT. FDG-PET/CT can differentiate a metabolic active tumor from atelectasis (15). In addition, RT target volume can also be determined by detecting necrotic regions in the tumor. Thus, maximum radiation can be applied to different regions within the tumor by adjusting the intra-target dose, while decreasing the radiation dose that peripheral tissues will receive. As a result, fibrosis and long-term side effects will decrease (16). Patients can be operated if there is no lymph node involvement (N0) or if there is only hilar lymph node involvement (N1) in NSCLC. SBRT provides good local control in non-operable patients with T1-2 tumor and without lymph node metastasis (N0) (80 Gray in 6-8 fractions; 30-60 Gy in 3-5 fractions) (17). For this reason, the highly accurate mediastinal staging provided by FDG-PET/CT is critical for early stage NSCLC patients who are candidates for SBRT. Li et al. (17) evaluated 200 patients by FDG-PET/CT before the operation in their multi-centered study. They compared PET and the histopathologic results of surgery specimens. The sensitivity rate (83%) and negative predictive value (NPV) (91%) of PET/CT were found to be very high for mediastinal lymph node staging. Treatment volumes change owing to the presence of non-enlarged lymph nodes with increased FDG uptake. Hellwig et al. (18) reported the sensitivity rate of CT and FDG-PET as 56% and 83% for all stages, respectively. Enlarged nodal size on CT showed a sensitivity of PET of 90% while sensitivity was 70% in normal lymph node sizes on CT. Routine elective nodal radiation is not recommended due to the high NPV of FDG-PET/CT in detecting mediastinal lymph node metastasis (19). SBRT can be a treatment option for patients if mediastinal lymph node metastasis is not detected in FDG-PET study. Selective lymph node radiation, which refers to the irradiation of lymph nodes with increased FDG-PET uptake, is a reliable method that provides local control in lymph nodes with decreased target volumes (16,20). Hwangbo et al. (21) found that mediastinal staging by FDG-PET/CT led to false positive results in approximately 30% of patients. Therefore, pathologic evaluation with mediastinoscopy or endoscopic ultrasound (EUS-FNAB) guided fine needle aspiration biopsy may be more suitable. The sensitivity of EUS-FNAB and that of FDG-PET/CT are similar in the detection of mediastinal metastasis (EUS-FNAB: 97.9%, FDG-PET: 96.3%) in squamous cell carcinoma while EUS-FNAB has a higher sensitivity in adenocarcinoma patients (EUS-FNAB: 94.6%, FDG-PET: 77.8%) (21).

A dosimetric study of van Der Wel et al. (20) evaluating 21 patients with N2 and N3 NSCLC showed that using FDG-PET/CT in radiation treatment planning process the esophagus and the lungs could be kept in the low dose area while the tumor received a high dose. FDG-PET/CT is one of the most important methods for the detection of NSCLC patients who are candidates for definitive RT. FDG-PET/CT affects staging by detection of distant metastasis and locally advanced disease, thus improving the success rates of cancer treatment with curative intent such as RT and chemotherapy (22). In a prospective study by Mac Manus et al. (23) on 153 patients, FDG-PET/CT changed treatment plan in 30% of patients to palliative treatment who were initially planned for curative-intent RT by conventional staging. This alteration was due to the detection of distant metastasis and extensive intra-thoracic disease in 20% and 10% of patients, respectively. Staging with PET predicts life expectancy more accurately than conventional staging in patients planned for curative-intent definitive RT. It allows prevention of unnecessary treatment for patients with a short life expectancy. The accuracy of staging with PET/CT in patients with NSCLC is higher and it allows radiation oncologists to treat the malignant tissue alone. In a prospective study including 105 NSCLC patients, the treatment strategy for 26% of the patients was changed from curative therapy to palliation after staging with FDG-PET, overall, the treatment plan was changed in 67% of the entire patient group (24). In another study including 153 NSCLC patients, it was observed that disease staging changed in 33% of the patients and target volumes changed in 25% of the patients after use of FDG-PET (25). It was further shown that the use of FDG-PET study for the detection of tumor volumes significantly decreased the differences in contouring between radiation oncologists. FDG also led to intra-observer changes, as the same oncologist contoured the same target differently when also using FDG PET (26).

## DEFINITION OF VOLUMES DELINEATED IN RADIOTHERAPY PLANNING

**Figure 1:**
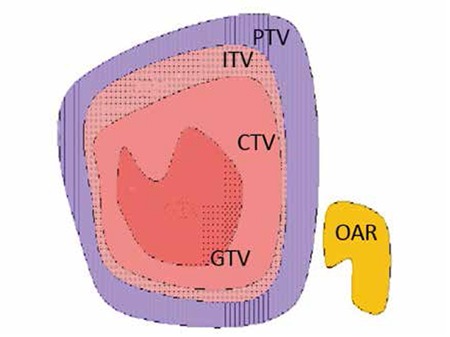
International Commission on Radiation Units 62: Treatment volume definitions PTV: Planning target volume, CTV: Clinical target volume, GTV: Gross tumor volume

The definitions related to tumor localization were described in International Commission on Radiation Units (ICRU) 29^th^, 50^th^, 62^n^d, 71^st^ and 83^rd^ reports in detail ICRU. In this study, the definition of target volumes was made by using these protocols (Figure 1).

### Gross Tumor Volume GTV:

The visible tumor volume that can be felt by hand and can be detected by methods such as CT or MR.

### Clinical Target Volume:

In various studies, it was shown that there were undetectable subclinical malignant cells around the gross tumor. Thus, it was considered that these areas should also be included in the treatment volume. The treatment volume is determined by the radiation oncologist based on the aim of treatment (cure versus palliation).

CTV=GTV + subclinical disease area

### Planning Target Volume:

Patient and machine factors are considered in the delineation of the planning target volume (PTV), which is a geometrical definition. Accurate selection of radiation areas and sizes in order to create the desired dose in CTV depends on the definition of PTV by considering error margins. PTV volume and shape are determined by the selected therapy techniques such as SBRT, tumor location and set-up errors that depend on the previously defined CTV. In addition, PTV is dependent on tumor location that can influence assessment with respiration or digestive organ movement such as peristalsis, as well as on patient set-up during treatment planning session and beam inaccuracies. The required confidence margins should be added to therapy plan to minimize these problems. Internal margins include physiological alterations in position, volume and shape of the tumor according to anatomical reference points (i.e. bladder and rectum become full differently in each treatment, respiration, intestine and heart movements). Set-up margin includes patient-specific clinical and instrument specific mechanical and dosimetric factors besides unavailability of the same position for the patient and radiation area according to coordinate systems of therapy instrument.

PTV=CTV + internal margin (IM) + set-up margin (SM).

### Internal Target Volume:

This is defined both in ICRU 62 and ICRU 50, and includes respiration, digestion, heart and other organ movements in addition to CTV.

ITV=CTV+IM

### Planning Organ at Risk Volume:

Therapy planning and total dose should be also decided by considering radiation sensitivities of critical organs and early/late side effects. Side effects can manifest in the long-term in some organs such as the spinal cord, and permanent damage can occur with high doses. Early side effects such as mucositis and diarrhea may lead to interruption of therapy. Generally, the dose limits of specific tissues and organs that can be tolerated without side effects should be determined and these doses should not be exceeded. IM and SM should be added while determining “planning organ at risk volume” (PVR) that can affect the dose area and therapy planning significantly.

PRV=OAR+IM+SM

## EFFECT OF FLUORODEOXYGLUCOSE POSITRON EMISSION TOMOGRAPHY ON TARGET VOLUME DETERMINATION

FDG-PET/CT leads to significant alterations in target volume size and shape as shown in various studies (15,20,27,28,29,30,31,32,33).

**Figure 2:**
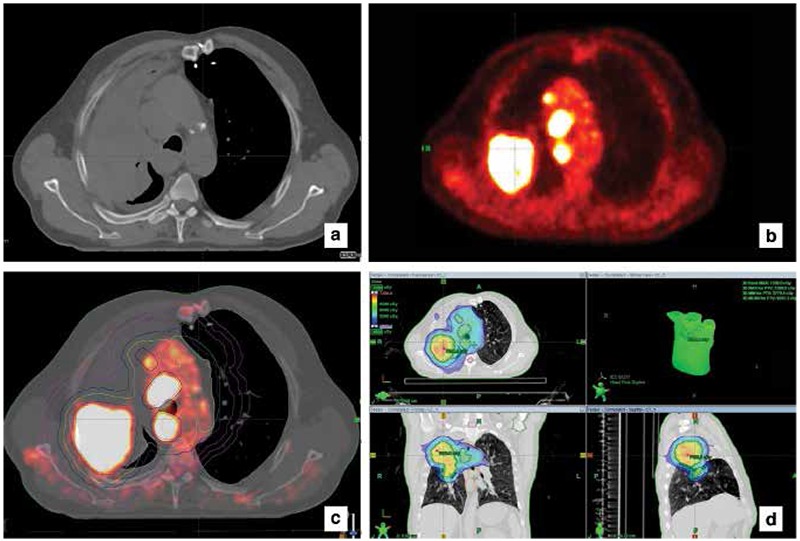
Non-small cell lung cancer, a) The atelectatic field cannot be separated from the tumor tissue in computed tomography, b) Positron emission tomography images showed increased fluorodeoxyglucose uptake in the tumor tissue, c and d) It was observed that there was a significant difference in target volumes formed by positron emission tomography/computed tomography fusion images

**Figure 3:**
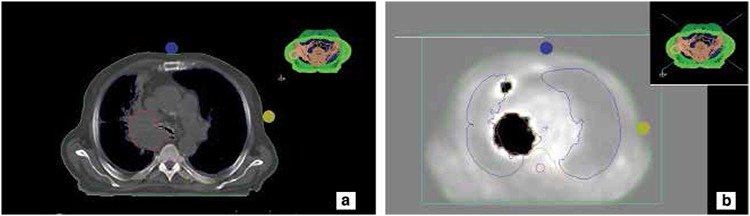
a) A normal-sized mediastinal lymph node in computed tomography images, b) Increased activity uptake was seen in positron emission tomography images, gross tumor volume included

In a recent review reported by Chi and Nguyen (34), it was observed that target volumes changed more than 20% while staging changed 20-50% as a result of treatment planning with FDG-PET. The most significant alteration was related to the differentiation of atelectasis from tumor tissue in PET images (Figure 2), and detection of lymph node metastases by observing increased FDG uptake in small-sized lymph nodes on CT (15,27,28,29,30,33) (Figure 3). Bradley et al. (29) showed that there was a 58% change in the delineation of tumor volume when planned using FDG-PET/CT in stage I-III NSCLC patients along with a 31% change in disease stage. The ability to identify and differentiate atelectasis led to a decrease in GTV delineation in 3 out of 24 patients planned for 3D conformal RT, while the ability to identify small lymph node metastases via high FDG uptake led to a GTV increase in 10 patients as well as detecting additional parenchymal disease in one patient. Furthermore, the dosage to the normal lung and esophagus decreases with a small GTV by excluding atelectasis. Similar studies showed that doses to the heart, esophagus, spinal cord and normal lungs decreased due to alterations of the target volume when using FDG-PET/CT (27,28,30,31,33). Although an increase in dose to peripheral tissue was observed in patients with greater GTV volume due to mediastinal lymph nodes detected by FDG-PET/CT, this increase was not found to be clinically significant in all patients. In a study of van Der Wel et al. (20), it was shown that nodal GTV decreased and thus, the dose that esophagus and normal received took decreased by FDG-PET/CT in N2-3 NSCLC patients. In a study performed in our clinic including 25 patients with lung cancer, a change was detected in 96% of the patients when target volumes were delineated by using F18 FDG-PET/CT versus CT alone. GTV and CTV volumes delineated by using FDG-PET/CT were lower than the volumes obtained by CT alone in 64% of the patients. This was due to PET/CT’s enabling differentiation of the tumor from atelectasis in the lung (35).

## DELINEATION OF TARGET VOLUMES BY FLUORODEOXYGLUCOSE POSITRON EMISSION TOMOGRAPHY

Once PET and CT images are obtained and fused, tumor and target volume delineation are the most important steps to follow. Individual view assessment for each patient and determination of tumor contours are required because of the differences in bio-distribution, dynamic and screening features of screening agents used in nuclear medicine. Thus, a standard use and algorithm of PET for the detection of target volumes is not available. Accurate and consistent detection of target volumes by PET is affected by certain factors. The first one is the limited spatial resolution of PET for the detection of GTV (it is approximately 4.5 mm in the latest generation PET/CT scanners) and partial difficulty in the determination of lesions due to poor resolution. The small lesions can be detected if only they have high FDG uptake while almost all of the lesions larger than 1 cm or those with increased FDG uptake 4 times greater than background activity can be detected. The detection of tumor contours by visual assessment is subjective, and hence proper delineation varies according to physician experience; certain lesions can be obscured due to partial poor spatial resolution. In addition, the proper assessment may be affected by view window selection, color scale, lesion/background ratio and high uptake in neighboring normal structures in PET images. These problems can be minimized by fusing PET and CT images. The tumor can be detected more clearly by obtaining more information with fused images of PET and CT imaging. PET images can also be used for RT planning in patients with FDG-PET staging and who are suitable for RT with curative intent (29). Ideally, PET staging images of RT candidates can be used directly in treatment planning if the views are acquired with the patient in the treatment position with suitable immobilization (36). If there is no PET/CT instrument in the department, PET and CT image fusion can be performed afterward, only if reference markers were used (37). If PET imaging are not acquired in the treatment position (i.e. if arms are not above the head) or if there has been a significant time lapse since staging PET images, then it is recommended that PET should be repeated in the accurate position. Target volumes can be delineated by visual assessment, experience and initiative of the physician as well as mathematical modeling methods using PET data obtained by semi-quantitative calculations in RT planning. Methods used for the detection of target volumes are as follows:

### 1. Visual Assessment

It is observed that standardized uptake value (SUVmax) and other similar parameters are being used in almost all studies in which RT planning is performed by visual assessment. Visual method is not defined in the literature, thus, it is recommended that a detailed protocol should be outlined by the centers that will use this method. Anatomic labels should confirm the suitability of PET/CT and fusion images, and the radiation oncologist and the nuclear medicine specialist should select the suitable diagnostic window before beginning the RT planning process by visual assessment. In a study by Doll et al. (38) including 44 international and different disciplines, it was shown that tumor volumes were determined most accurately in teams including nuclear medicine doctor and radiation oncologist.

### 2. Automatic or Semi-Automatic Methods

Methods that are more objective were investigated using automatic and semi-automatic methods in order to decrease the inter-user variability in the detection of target tumor volumes by FDG-PET/CT. However, these methods could not differentiate neoplastic tissue from physiologic and inflammatory processes since FDG is not a tumor-specific substance. Tumor volumes required revision in studies using real patient data while these methods yielded good results in studies using phantom data. It should be remembered that FDG is involved in macrophages and granulation tissue beside tumor cells. FDG-PET is a map showing three-dimensional glucose distribution, but it is not a map showing cancer cells (39).

### 2.1. Standardized Uptake Value

SUVmax is the most compatible and reliable quantitative parameter commonly used for the assessment of tumor activity in clinical practice [SUVmax: Maximum activity concentration/(injected dose/weight)]. Eighty-seven patients with pulmonary nodules were included in the study. Lesions were confirmed by pathological assessment and followed up for at least 2 years. When the threshold value for SUV was considered as 2.5 for the diagnosis of lung cancer, the sensitivity, specificity and accuracy were 97%, 82%, and 92%, respectively (40). Therefore, SUV threshold value is recommended as 2.5 for the detection of GTV during RT planning (41).

### 2.2. Thresholding Method

In the most common thresholding approach, it is accepted that selection of the field with constant percent uptake levels according to maximum activity value of tumor helps to determine tumor contour (42). In the studies in which constant threshold value was accepted as 40-50%, it was observed that this threshold value led to serious errors in lesion-size, homogeneity, and lesion/background contrast-depended volume calculations (43). This approach was shown to decrease GTV significantly in primary NSCLC patients who showed large, non-homogeneous activity uptake by comparing various contouring methods (44). Thus, more studies are required for the detection of gross tumor contours by contrast-dependent adaptive thresholding methods.

### 2.3. Background Cut-off Method and Source/Background Plan Algorithms

 In the background cut-off method, which is another automatic contouring method, tumor volumes are formed by drawing the field above the detected value (i.e. the fields showing 3 standard deviations from background activity for increase uptake level, fields above 2.5 SUV) (45). The advantage of this method is the detection of contours separately from heterogeneous FDG uptake in the lesion. However, the accuracy of this background cut-off approach depends on the accuracy of the statistical method used for this method. Contrast-oriented thresholding algorithm is obtained by calculating the effects of background FDG concentration on tumor volumes for the detection of GTV by PET in NSCLC patients (46). This approach showed that the GTV decreased as compared to the volumes obtained by CT alone, and it was compatible with pathologic tumor volume. This study detected a significant difference in pathologic tumor volume for tumors located in the lower lobes, as a result of breathing motion. It was predicted that these mistakes can be prevented by three-dimensional PET imaging. In a study, in which source/background ratio determined by auto-segmentation approach was used, the results were compatible with pathologic tumor size in 33 NSCLC patients (47).

### 2.4. Gradient-Based Approach

PET-GTV detected by gradient-based approach is recommended in order to minimize statistical image noise and resolution blur (48). In a phantom study by Werner-Wasik et al. (49), gradient-based approach produced results that were more accurate as compared to other methods in terms of PET-GTV detection. In addition, this method was also compared with other methods in which GTV was determined by comparing to surgical samples (50,51). A study including 10 patients who had undergone lobectomy for stage I-II NSCLC found that PET-GTV detected by 40-50% constant threshold and source/background ratio methods was better than GTV detected visually on CT images (50). In another study including 19 patients, tumor volumes detected by the gradient-based approach in PET/CT images obtained during normal breathing before surgery were highly compatible with surgical pathologic results (51).

### 2.5. Automatic Methods

Full-automatic thresholding methods were developed for the detection of tumor volumes by FDG-PET in lung cancer patients. Automatic thresholding methods using source/background algorithm is one of the most frequently used methods. If automatic contouring is performed using only functional images for RT planning, serious errors may occur. Primarily, it is important to match the images obtained by CT and the other anatomic imaging methods before determining tumor volume by automatic segmentation. Afterward, these should be revised by the planner since pathologic and physiologic distribution of the radioactive substance cannot be differentiated by automatic thresholding methods.

IA standardized automatic method for the delineation of GTV has not been established yet, although the methods discussed above have been reported for the detection of tumor volume. The concordance of real tumor volume obtained by surgery and different GTV detection methods in NSCLC patients are summarized in Table 1 (34).

## TUMOR MOVEMENT: RADIOTHERAPY PLANNING BY GATED POSITRON EMISSION TOMOGRAPHY/COMPUTED TOMOGRAPHY

Organ movement and thus, tumor motion due to respiration, cardiac cycle, and other factors, is a significant issue in the delineation of the target tumor volume in thoracic malignancies. Calculations in RT planning should be performed by taking organ movements into consideration (56). A more accurate treatment can be delivered by following organ movement. If the tumor volume moves out of the contours, a part of the tumor can remain in the low-dose field due to organ motion. A few non-randomized studies described adverse effects related to average lung dose, despite the belief that high doses are advantageous for lung cancer patients (57). The peripheral healthy tissue can be preserved while increasing the dose to the tumor, and a smaller margin can be used to create the PTV by four-dimensional (4D) RT. PET, as well as CT images, can be recorded synchronized with the respiratory cycle in 4D Gated PET/CT imaging. Images are formed by taking a specific phase of 4D PET/CT respiration cycle as a reference point, and image of the tumor that moves with breathing can be obtained (58). Therefore, tumor contours can be delineated more accurately and normal tissues can be preserved better. 4D Gated PET/CT corrects for movement-depended motion blur and shows the functionally active field of the tumor that is mobilized with respiration more clearly. In a study by Lamb et al. (59), tumor volumes of 4 lesions in the lower lobes of 3 patients were delineated, calculated by both 4D CT and 4D Gated PET/CT, and were compared. In this study, GTVs obtained by 4D PET/CT were 30% smaller in volume than those obtained by 4D CT. In the same study, the difference between the target tumor volumes obtained by 4D CT and normal PET images was deemed as minor. Gated PET allows more accurate GTV detection than 4D CT for SBRT planning, especially in tumors located in the lower lobes, which have more movement. 4D CT has a decreased accuracy of tumor motion assessment in lower lobe lesions, due to the proximity of the tumor to soft tissues such as the liver on the right and the spleen and stomach on the left. Tumor motion is the most significant obstacle to the planning of conformal RT. The treating system should be compatible with the differences due to tumor motion (real time monitoring) at all times (60) or the radiation dose should be delivered only at a specific phase of the respiratory cycle. As a result, the definition of three-dimensional conformal RT is termed as a four-dimensional or gated radiation therapy.

## CLINICAL RESULTS OF RADIOTHERAPY PLANNING PERFORMED BY POSITRON EMISSION TOMOGRAPHY/COMPUTED TOMOGRAPHY

**Figure 4:**
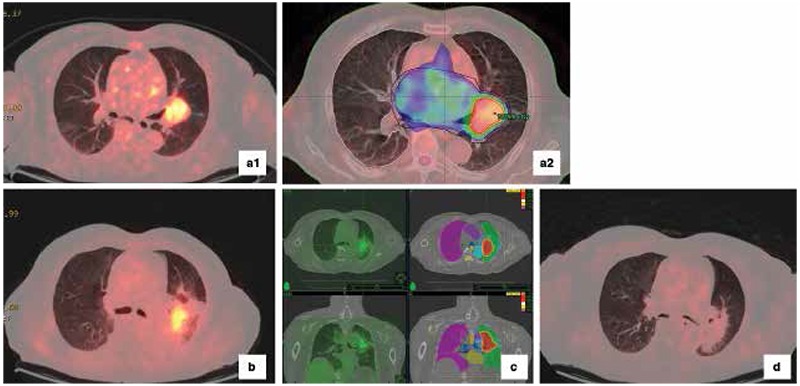
Non-small cell lung cancer, 82 y.o., M patient, a1) Abnormal increased metabolic activity was observed in the mass identified in the left parahilar region and, a2) Intensity modulated radiation therapy planning images by these views, b) Increased metabolic activity compatible with residual mass was seen in positron emission tomography/computed tomography that was applied for treatment-control after four months, c) Plan images belonged to stereotactic body radiation therapy that was applied to the residual mass, d) Pathologic activity was not observed in positron emission tomography/computed tomography after one year (complete metabolic response)

The literature indicates that FDG-PET/CT significantly decreases clinical tumor volumes in patients with large lymph nodes without FDG uptake and atelectasis due to its high diagnostic accuracy in NSCLC. FDG-PET/CT was also observed to have a prognostic value since the SUVmax value is reported to predict survival in primary NSCLC patients. In addition, the pre- and post-RT SUVmax values were found to correlate with overall survival and disease-free survival. High SUVmax values were associated with poor survival in primary lung tumors and with the presence of lymph node metastasis (61). High glucose uptake of the tumor is related to its high metastatic potential. Mac Manus et al. (62) investigated the role of FDG-PET in the assessment of response to RT. In this study, screening was performed by FDG-PET for 88 NSCLC patients before and after chemotherapy (on average 70 days after RT initiation, 60 Gy, 30 fractions for 6 weeks). The complete metabolic response was obtained in 45% of the patients while a partial metabolic response was obtained in 36% of the patients by FDG-PET after treatment. The mean survival was 31 months and 11 months in the group with a complete metabolic response and in the group without response, respectively. One-year survival was determined as 93% and 47% in the group with complete response and without response, respectively. This study detected that the results were statistically significant despite confounders such as inflammation due to RT. There are significant differences in SUVmax changes during RT between the group that showed metabolic response and the group with no response. Overall survival was higher in the group with a metabolic response. The decrease ratio in SUVmax value directly correlated with disease-free survival (63). FDG-PET/CT plays an important role in the detection of recurrent disease. Metabolic evaluation by PET/CT in NSCLC has a high accuracy rate after treatment (78-98%) (64). Investigations can be performed as early as 6 weeks post treatment due to pneumonia and inflammation that may occur after RT, but the recommended interval is frequently 3-6 months. Metabolic response assessment is an important parameter for the detection of local failure and survival. The detection of residual metabolic activity allows for the possibility of additional planning (Figure 4). In a study by Velasquez et al. (65), PET images were assessed before and after radical chemotherapy in 101 non-operated NSCLC patients. The overall survival was significantly decreased in the group showing residual metabolic uptake in the post-treatment PET study. There was an association between detection of residual increased metabolic uptake after RT in large tumors and tumors with high SUVmax value. The clinical results of stage II-III NSCLC patients whose disease volumes were identified by FDG-PET/CT and who were treated with RT were reported in two recent studies (66,67). In a pilot study including 32 patients treated with RT, only one local failure and one local progression were detected (66). The recurrence was in a lymph node. When the treatment plans were revisited, it was observed that this lymph node was FDG positive but was not included in the treatment volume. In another study on 137 stage III NSCLC patients and PET-positive areas, local recurrence was reported as 14.6% and distant metastases as 16.8% (67). These findings showed that clinical results of patients with stage II and III disease whose RT were planned by PET were as good as of those planned by using CT (68). Furthermore, the dose to the primary lesion can be increased while preserving the normal tissue in locally advanced NSCLC by using PET. Therefore, local control can be increased as well as survival, as was suggested by Aupérin et al. (69) in a meta-analysis.

## RADIOTHERAPY PLANNING BY FLUORODEOXYGLUCOSE POSITRON EMISSION TOMOGRAPHY/COMPUTED TOMOGRAPHY IN SMALL CELL LUNG CANCER STAGING

SCLC consists of approximately 20-25% of lung cancers. It is often diagnosed at an advanced stage with distant metastases and exhibits an aggressive clinical behavior (70). In spite of aggressive treatment, it carries a poor prognosis (9). Accurate staging in SCLC is the most important factor in determining the most appropriate method of treatment. It is difficult to determine disease spread and especially to assess the involvement of mediastinal lymph nodes. Fischer et al. (71) compared FDG-PET/CT as a staging tool with standardized staging modalities (CT and bone scintigraphy) in their prospective study including 29 SCLC patients. In this study, PET/CT changed the plan in five of 29 patients (17%), and it was shown that this method increased the accuracy of tumor definition. In another study, 8.3-9.5% of patients were up-staged to advanced stages with the addition of FDG-PET/CT (72,73). Arslan et al. (74) evaluated the accuracy of staging by FDG-PET/CT and CT, and its relation to overall survival. When compared to staging by CT, staging by FDG-PET/CT up-staged nine of 25 patients(36%), while down-staging two patients (8%). In addition, a significant survival difference was predicted (p=0.019) by using FDG-PET/CT in the staging process, but there was no difference in those in which CT had been used (p=0.055). These studies recommend the use of FDG-PET/CT for initial staging in SCLC during limited stage.

## DETERMINATION OF TUMOR VOLUMES BY FLUORODEOXYGLUCOSE POSITRON EMISSION TOMOGRAPHY/COMPUTED TOMOGRAPHY

There are less studies in which tumor volumes were determined by FDG-PET/CT in SCLC patients when compared to NSCLC. Nevertheless, it can contribute to the treatment planning process by determining tumor volumes more accurately as in NSCLC.

## RADIOTHERAPY PLANNING FOR THE FIELD INVOLVED LYMPH NODE WITH FLUORODEOXYGLUCOSE POSITRON EMISSION TOMOGRAPHY/COMPUTED TOMOGRAPHY

Elective nodal radiation of mediastinal lymph nodes in SCLC patients with limited disease decreases nodal failure rate. However, some physicians are reluctant to offer elective nodal radiation due to serious adverse effects as a result of large field of radiation. Baas et al. (75) evaluated involved field RT in early stage SCLC patients diagnosed by CT in their phase II study. Mean survival was 19.5 months with acceptable adverse effects. De Ruysscher et al. (76) investigated involved field RT in early stage SCLC patients diagnosed by CT in their phase II study. They assessed general survival and isolated lymph node failure rates, which was described as relapse in local lymph nodes out of target volumes in patients who had no failure identified in the treatment field. In this study, isolated lymph node metastases that were not included in the treatment field was found to be unexpectedly high (11%). Involved field RT was considered as controversial by International Atomic Energy Agency (IAEA) when these findings were evaluated, and further prospective clinic studies were suggested (77). FDG-PET/CT can be used in order to determine the requirement of elective nodal radiation. Two recent studies assessed the requirement for elective nodal radiation after staging by FDG-PET/CT (78,79). In a prospective study, van Loon et al. (78) assessed involved field RT by FDG-PET in 60 patients with limited stage SCLC disease. The mean overall survival was 19 months and isolated nodal failure rate was 3%. The isolated nodal failure rate was significantly lower in the group planned by FDG-PET as compared to the group planned by CT (11% vs. 3%). Shirvani et al. (79) evaluated 60 patients with limited stage SCLC who were staged by FDG-PET and were treated with IMRT involved field RT. The 2-years survival rate was calculated as 58%, and isolated nodal failure was detected in one patient (3%). These studies concluded that involved field RT could be used reliably instead of elective nodal radiation in patients staged by F18 FDG-PET. In this way, the toxicity can be decreased or adjusted by not irradiating the PET negative lymph nodes. Involved field RT by FDG-PET in SCLC is a current discussion subject and the use of involved field RT instead of elective nodal radiation should be assessed by further prospective studies. Another role of FDG-PET in SCLC is the evaluation of response to treatment. It was found that assessment success of FDG-PET in patients who received chemotherapy and RT was high (80).

## CONCLUSION

In conclusion, the most important contribution of FDG-PET to the management of SCLC is in the accuracy of staging. Although RT use with the help of FDG-PET in this patient group is controversial, involved field radiation is an attention-grabbing method. FDG-PET treatment planning can change treatment strategy of SCLC patients with limited disease.

## Ethics

Peer-review: Externally peer-reviewed.

## Figures and Tables

**Table 1:**
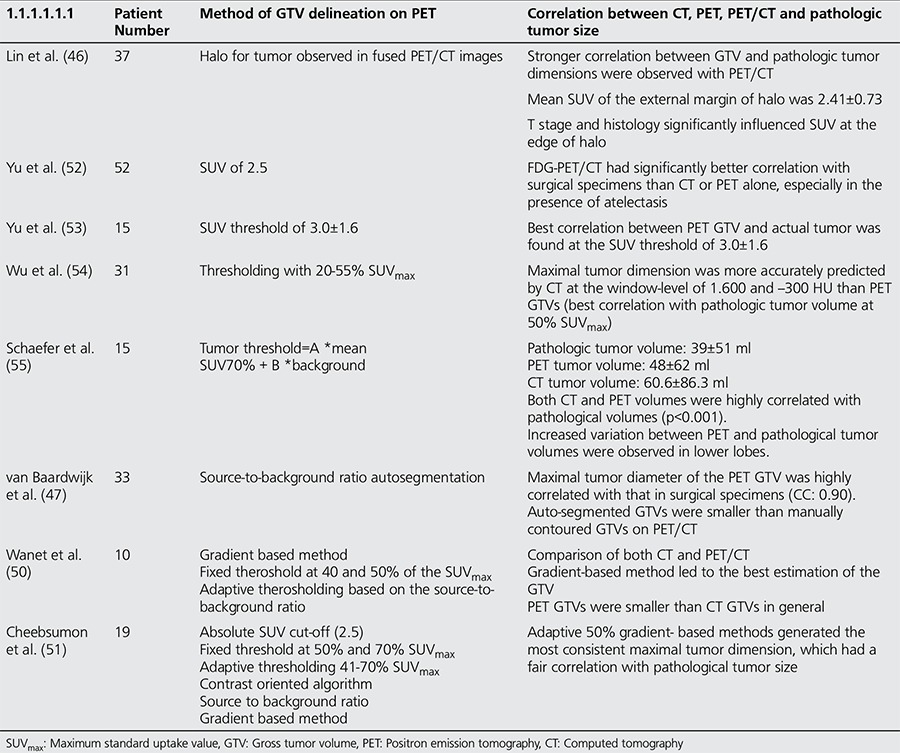
Methods of gross tumor volume delineation on positron emission tomography in correlation with surgical specimens
